# A new methodology to derive 3D kinetic parametric FDG PET images based on a mathematical approach integrating an error model of measurement

**DOI:** 10.1186/s13550-018-0454-9

**Published:** 2018-11-15

**Authors:** Elyse Colard, Sarkis Delcourt, Laetitia Padovani, Sébastien Thureau, Arthur Dumouchel, Pierrick Gouel, Justine Lequesne, Bardia Farman Ara, Pierre Vera, David Taïeb, Isabelle Gardin, Dominique Barbolosi, Sébastien Hapdey

**Affiliations:** 1 LITIS-QuantIF-EA4108, University of Rouen, Rouen, France; 2 Department of Nuclear Medicine, La Timone University Hospital, Marseille, France; 3 Department of Radiotherapy, La Timone University Hospital, Marseille and SMARTc-INSERM-UMR 911 CR02, Aix-Marseille University, Marseille, France; 4 Department of Radiotherapy, Centre Henri Becquerel, Rouen and LITIS-QuantIF-EA4108, University of Rouen, Rouen, France; 5 Department of Nuclear Medicine, Centre Henri Becquerel, Rouen, France; 6 Department of Clinical Research, Centre Henri Becquerel, Rouen, France; 7 Department of Nuclear Medicine, La Timone University Hospital, Marseille and CERIMED, Aix-Marseille University, Marseille, France; 8 Department of Nuclear Medicine, Centre Henri Becquerel, Rouen and LITIS-QuantIF-EA4108, University of Rouen, Rouen, France; 9 SMARTc–CRCM, INSERM UMR1068, CNRS UMR7258, Aix Marseille Université U105, Institut Paoli Calmette et APHM, Marseille, France

**Keywords:** Dynamic PET, Parametric imaging, FDG kinetics, Lung cancer, Quantification

## Abstract

**Background:**

In FDG-PET, SUV images are hampered by large potential biases. Our aim was to develop an alternative method (ParaPET) to generate 3D kinetic parametric FDG-PET images easy to perform in clinical oncology.

**Methods:**

The key points of our method are the use of a new error model of PET measurement extracted from a late dynamic PET acquisition of 15 min, centered over the lesion and an image-derived input function (IDIF). The 15-min acquisition is reconstructed to obtain five images of FDG mean activity concentration and images of its variance to model errors of PET measurement. Our approach is carried out on each voxel to derive 3D kinetic parameter images. ParaPET was evaluated and compared to Patlak analysis as a reference. Hunter and Barbolosi methods (Barbolosi-Bl: with blood samples or Barbolosi-Im: with IDIF) were also investigated and compared to Patlak. Our evaluation was carried on *K*_i_ index, the net influx rate and its maximum value in the lesion (*K*_i,max_).

**Results:**

This parameter was obtained from 41 non-small cell lung cancer lesions associated with 4 to 5 blood samples per patient, required for the Patlak analysis. Compare to Patlak, the median relative difference and associated range (median; [min;max]) in *K*_i,max_ estimates were not statistically significant (Wilcoxon test) for ParaPET (− 3.0%; [− 31.9%; 47.3%]; *p* = 0.08) but statistically significant for Barbolosi-Bl (− 8.0%; [− 30.8%; 53.7%]; *p* = 0.001), Barbolosi-Im (− 7.9%; [− 38.4%; 30.6%]; *p* = 0.007) or Hunter (32.8%; [− 14.6%; 132.2%]; *p* < 10^− 5^). In the Bland-Altman plots, the ratios between the four methods and Patlak are not dependent of the *K*_i_ magnitude, except for Hunter. The 95% limits of agreement are comparable for ParaPET (34.7%), Barbolosi-Bl (30.1%) and Barbolosi-Im (30.8%), lower to Hunter (81.1%). In the 25 lesions imaged before and during the radio-chemotherapy, the decrease in the FDG uptake (ΔSUV_max_ or Δ*K*_i,max_) is statistically more important (*p* < 0.02, Wilcoxon one-tailed test) when estimated from the *K*_i_ images than from the SUV images (additional median variation of − 2.3% [− 52.6%; + 19.1%] for Δ*K*_i,max_ compared to ΔSUV_max_).

**Conclusion:**

None of the four methodologies is yet ready to replace the Patlak approach, and further improvements are still required. Nevertheless, ParaPET remains a promising approach, offering a non-invasive alternative to methods based on multiple blood samples and only requiring a late PET acquisition. It allows deriving *K*_i_ values, highly correlated and presenting the lowest relative bias with Patlak estimates, in comparison to the other methods we evaluated. Moreover, ParaPET gives access to quantitative information at the pixel level, which needs to be evaluated in the perspective of radiomic and tumour response.

**Trial registration:**

NCT 02821936; May 2016.

## Introduction

Positron emission tomography using 18-fluorodeoxyglucose (FDG PET) is useful for tumour staging, radiotherapy planning, treatment response and disease progression assessment [1].

FDG uptake is characterised by the standardised uptake value (SUV), which is widely used in clinical practice, based on a late static image acquired 60 min post-injection [2]. Therefore, different factors (both technical and physiological) can affect SUV calculation because it does not accurately represent the exact glucose metabolic rate, nor differentiate metabolised from unmetabolised FDG within the tumour [3]. Moreover, SUV is sensitive to patient preparation, scanning procedure, image reconstruction and image analysis procedures, compromising its use for pre-, per- and post-treatment scans comparison and inter-patient comparison [3, 4].

An alternative consists in the determination of the total behaviour of glucose within the lesion using more quantitative measurements. The gold standard for modelling tissue time-activity concentration curves derived from dynamic FDG PET acquisitions is the full kinetic analysis with compartmental modelling using a nonlinear least-squares regression [5] or the simplified Patlak graphical analysis [6]. These methods provide access to FDG kinetic parameters such as the net influx rate constant (*K*_i_). Several studies have demonstrated the potential added value of kinetic parameters over SUV measurement for the assessment of disease progression [7]. Despite Patlak rapid calculation and simple expression, the method has two major practical limitations: the need for continuous blood sampling and a 1-h dynamic acquisition. Several derived Patlak analyses using image-derived input function (IDIF) extracted from the images of the aorta or the left ventricular to create the shape of the FDG blood concentration function *C*_*p*_(t) have been proposed in the literature [8–10].

As an alternative to Patlak approach, several simplified quantitative methods have been proposed [11, 12], such as Hunter method based on a static acquisition and a venous blood sample to scale the tri-exponential blood activity curve for each patient. Recently, Barbolosi et al. [13] proposed a methodology to calculate global FDG kinetic parameters for the whole lesion, taking into account measurement errors. However, all these methods required blood samples.

In the literature, refined quantitative methods have been proposed to compute 3D kinetic parametric FDG PET images [13–15]. However, these methods required complex reconstruction algorithms and long PET acquisition that have prevented their clinical adoption.

Our aim was to develop a method to generate 3D kinetic parametric FDG PET images easy to perform in clinical routine. This method named ParaPET is based on previous work from Barbolosi et al. [13]. The main improvements of our method are the use of a new error model of PET measurement, an IDIF from a late dynamic acquisition and the measurement of kinetic parameters at the voxel level. This method is evaluated and compared to a derived Patlak analysis as a reference, and with Hunter and Barbolosi methods (Barbolosi-Bl: with blood samples or Barbolosi-Im: with IDIF).

## Material and methods

### Methods

To determine the FDG kinetic parameters within the lesion, the linear approximation of the mathematical representation of the standard three-compartment model with irreversible trapping (*k*_4_ = 0) is considered according to Patlak analysis [6]. From *C*_FDG_(*t*_k_), the FDG activity concentration in the lesion (Bq mL_tissue_^−1^) at a given time *t*_k_ after injection, the analytical solution of the three-compartment model verifies:

1 $$ {C}_{\mathrm{FDG}}\left({t}_k\right)={\mathrm{K}}_{\mathrm{i}}{\int}_0^{t_k}{C}_p(t) dt+{\mathrm{V}}_{\mathrm{p}}\ {\mathrm{C}}_{\mathrm{p}}\left({t}_k\right) $$

where *C*_p_(*t*_k_) represents the FDG activity concentration in blood plasma at time *t*_k_ (Bq mL_blood_^−1^) and *V*_p_ the total blood distribution volume (i.e. the unmetabolized fraction of FDG in blood and interstitial volume, *unitless*). *K*_i_, the net influx rate, is expressed as a combination of the compartmental transfer rates (*K*_*i*_ = (*k*_1_. *k*_3_)/(*k*_2_ + *k*_3_)). The *K*_i_ unit is mL_blood_ × mL_tissue_^−1^ × min^*−*1^.

#### Barbolosi approach

**Fig. 1:**
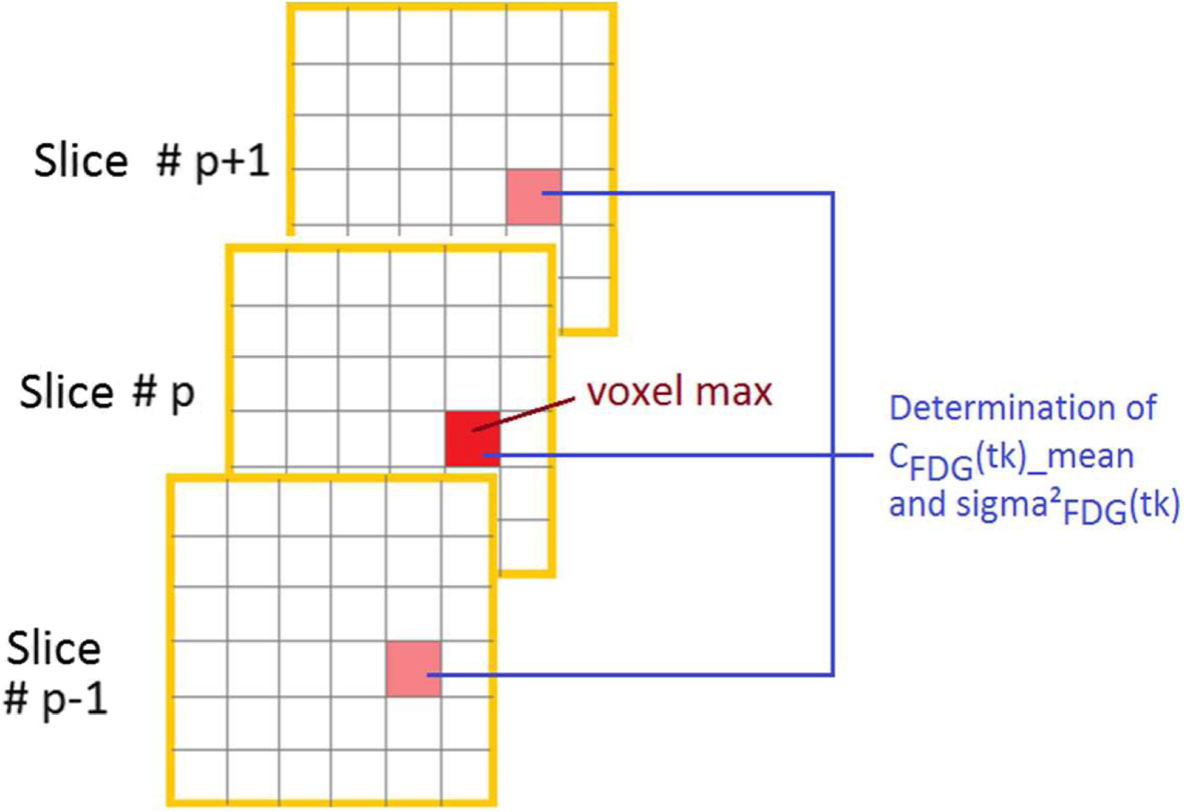
Barbolosi’s determination of $\overline{C_{\mathrm{FDG}}\left({\mathrm{t}}_{\mathrm{k}}\right)}$ and ${\upsigma}_{\mathrm{FDG},{t}_{\mathrm{k}}}^2$; The same voxel position is use for each study time *t*_k_

In this approach (further details in [13]), the *K*_i_ and *V*_p_ values are obtained by solving Eq. 1 for four time points *t*_k_, considering that *C*_FDG_(*t*_k_) and *C*_p_(*t*_k_) are incorrect estimates of the true FDG activity concentration in the PET images and blood draws. The measurement variability of *C*_FDG_(*t*_k_) and *C*_p_(*t*_k_) is taken into account, leading to the following mathematical expressions:

2 $$ {C}_{\mathrm{FDG}}\left({t}_k\right)=\overline{C_{\mathrm{FDG}}\left({t}_k\right)}+{\varepsilon}_{\mathrm{FDG},{t}_k} $$

3 $$ {C}_p\left({t}_k\right)=\overline{C_p\left({t}_k\right)}+{\varepsilon}_p $$

where $\overline{C_{\mathrm{FDG}}\left({t}_k\right)}$ is the mean maximum FDG activity concentration in the lesion, averaged over three consecutive 2D slices (see Fig. 1) and ${\varepsilon}_{\mathrm{FDG},{t}_k}$ the associated error, at study time *t*_k_. $\overline{C_p\left({t}_k\right)}$ is the mean FDG blood activity concentration measured from four blood samples, and *ε*_*p*_ the associated error derived from the counting efficiency which is considered identical for the same four time points *t*_k_. The experimental errors ${\varepsilon}_{\mathrm{FDG},{t}_k}$ and *ε*_*p*_ are distributed normally around 0 with ${\sigma}_{\mathrm{FDG},{t}_k}^2$ and ${\sigma}_p^2$, their respective variance [13].

For each time *t*_k_, 10,000 couples of (*C*_FDG_(*t*_k_), *C*_p_(*t*_k_)) are computed from 10,000 random draws of ${\varepsilon}_{\mathrm{FDG},{t}_k}$ and *ε*_*p*_ defined according to their respective distribution. Then, 10,000 couples of *x* and *y* values are obtained by minimising the following function:

4 $$ f\left(x,y\right)=\sum \limits_{i=1}^4{\left(x{\int}_0^{t_k}{C}_p\left(\tau \right) d\tau +{yC}_p\left({t}_k\right)-{C}_{\mathrm{FDG}}\left({t}_k\right)\right)}^2 $$

with 0 ≤ *x* ≤ *K*_H_ and 0 ≤ *y*, where *K*_H_ is derived from the Hunter model [12]. It corresponds to the estimation of *K*_i_ index at the first study time point *t*_1_ and is used as the maximal boundary in the search of *x*. A single couple of *K*_i_ and *V*_p_ values, corresponding to the mean values of *x* and *y*, respectively, is used to characterise the whole lesion.

#### ParaPET approach

Our proposed approach is a non-invasive extension of Barbolosi method at the voxel level, integrating a new error model of measurement, yielding to the determination of 3D parametric images of the lesion.

To improve the error model of measurements, we propose to compute five images of FDG mean activity concentration, associated with five images of its variability from FDG PET images centered over the lesion.

**Fig. 2:**
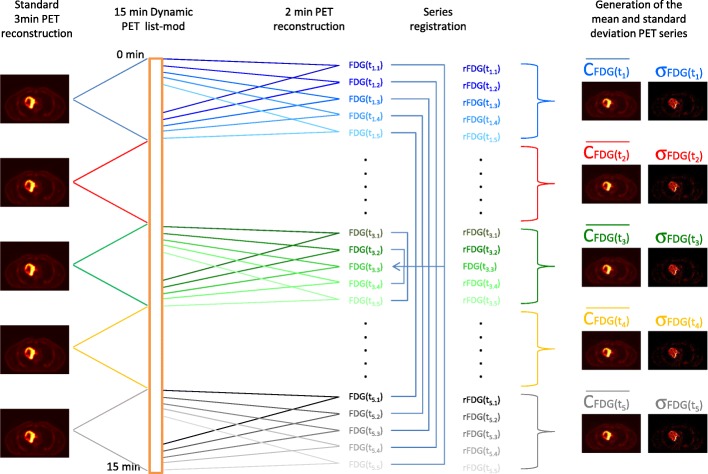
ParaPET reconstruction scheme. The 15 min dynamic acquisition is rebinned into five series of 3 min listmode datasets. For each 3 min listmode dataset, multiple reconstructions of 2 min are generated to derive $\overline{C_{\mathrm{FDG}}\left({t}_{\mathrm{k}}\right)}$ and ${\upsigma}_{\mathrm{FDG},{t}_{\mathrm{k}}}^2$ series

To that end, a delayed 15 min FDG dynamic PET acquisition performed at 80–90 min post-injection and centered over the lesion is necessary. This acquisition is resampled into five series of 3 min listmode datasets. For each 3 min listmode dataset, five reconstructions of 2 min are generated. For example, for the first 3 min listmode bin (*t*_1_), we reconstructed the data from 0 to 2 min, 15 s–2 min 15 s, 30 s–2 min 30 s, 45 s–2 min 45 s, and 1–3 min. From our 15 min listmode datasets, we finally generated 25 reconstructed series. This allows the determination of $\overline{C_{\mathrm{FDG}}\left({t}_k\right)}$ and ${\sigma}_{\mathrm{FDG},{t}_k}^2$ (*k* = 1 to 5) variables at the voxel level needed for Eq. 2 (cf Fig. 2).

Since we propose a methodology applicable at the voxel level, it is mandatory to avoid any misalignment between the 25 reconstructions used to derive $\overline{C_{\mathrm{FDG}}\left({t}_k\right)}$ and ${\sigma}_{\mathrm{FDG},{t}_k}^2$. A precise registration of the series must be made beforehand. In order to minimise the offset applied to each series, all data were locally realineated on the central time series (*k* = 3) using a 3D rigid registration method based on mutual information.

To avoid multiple blood samples, a late IDIF from FDG PET images is determined. Five regions of interest (ROI) are manually drawn over the aorta at each study time *t*_k_, allowing the extraction of the mean activity concentration $\overline{C_p\left({t}_k\right)}$ and its variance ${\sigma}_{p,{t}_k}^2$ needed for the error model in Eq. 3. The shape of the *C*_*p*_(*t*) function is modelled using the tri-exponential function developed in Hunter’s method.

Finally, the equation resolution following the approach previously proposed by Barbolosi (Eq. 4) is carried out on each voxel to derive parametric PET images of FDG kinetic parameters.

In practice, to perform our non-invasive ParaPET approach, only a late dynamic PET acquisition of 15 min performed at 80–90 min post-injection and centered over the lesion is necessary.

### Method evaluation

**Fig. 3:**
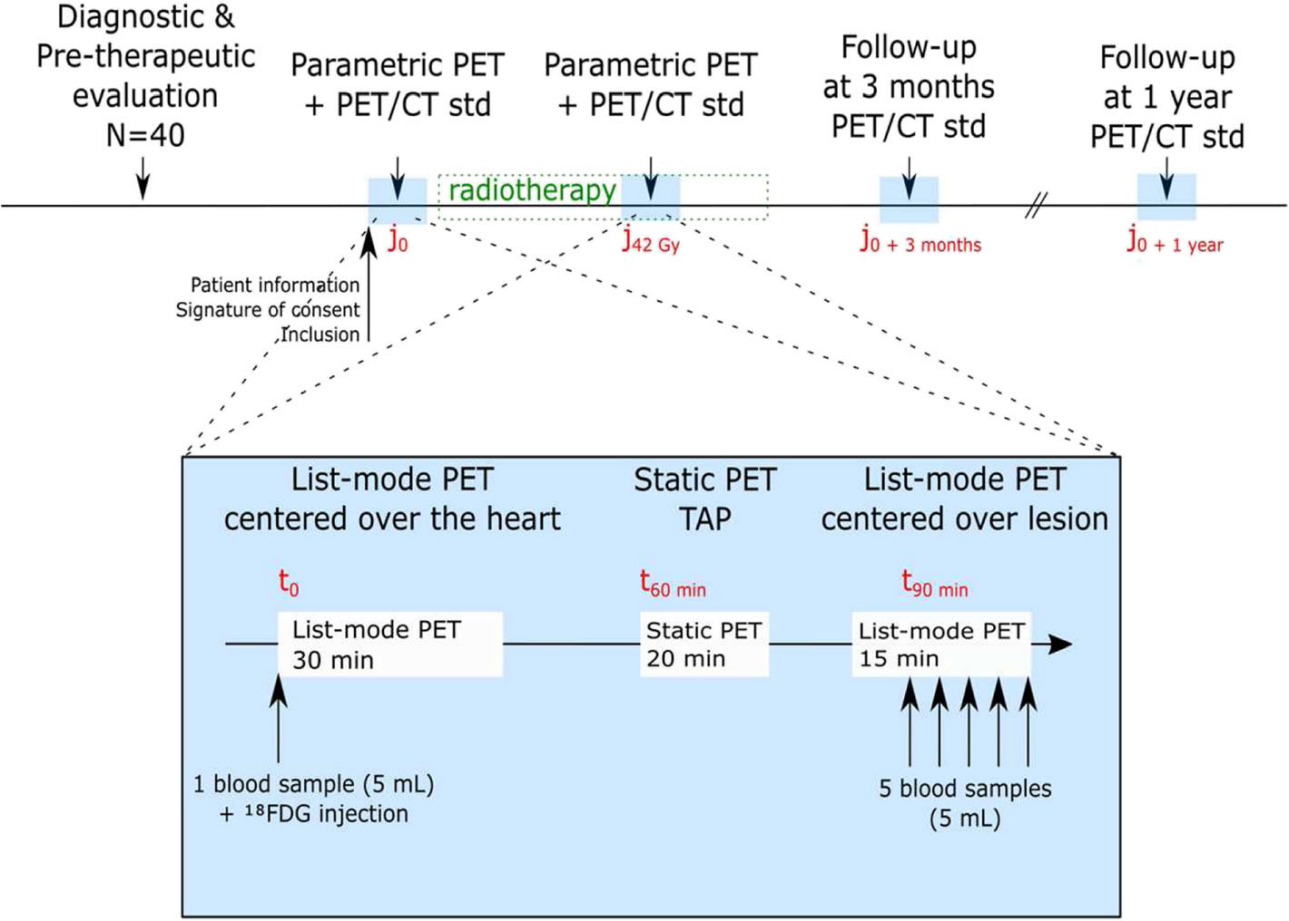
ParaPET clinical trial design. All patients benefit from two parametric acquisition sequences, one before and one at 42 Gy of their radiotherapy

Our method was evaluated on patients suffering for stage III non-small cell lung cancer (NSCLC) included in the ParaPET clinical trial (NCT 02821936). The study protocol was approved by the local Research Ethics Committee and has therefore been performed in accordance with the ethical standards laid down on the 1964 Declaration of Helsinki and its later amendments. All patients gave their written informed consent. Each patient benefits from 2 PET-CT exams, the baseline exam performed before any treatment and a per-therapeutic exam, around 42 Gy (21 days) of the radiotherapy, as indicated in Fig. 3.

All patients benefit from two parametric acquisition sequences, performed on a Discovery 710 PET/CT device (GE Healthcare, Milwaukee, WI, USA). The acquisition sequence is divided into three acquisitions (cf. Fig. 3):

1. An early 30 min listmode PET acquisition centered over the heart, starting simultaneously with FDG injection of 3.5 MBq/kg. This acquisition which is required for the Patlak analysis only, was reconstructed using a 30 frames protocol [16];
2. A 20-min standard whole-body PET acquisition was carried out 60 min post injection. These clinical routine images are not used in our protocol;
3. Lastly, a 15 min listmode PET acquisition centered over the lesion (performed 80–90 min post-injection). Five late venous blood samples are required for the implementation of the approaches of Hunter, Barbolosi and Patlak. The blood withdraws were realised each 3 min, starting 1.5 min after the beginning of the listmode acquisition.

Our evaluation was carried on *K*_i_ index, the net influx rate and its maximum value in the lesion (*K*_i,max_). We evaluated the robustness of our method with regards to the number of random draws of ${\upvarepsilon}_{\mathrm{FDG},{t}_k}$ and ${\upvarepsilon}_{\mathrm{p},{t}_k}$ by repeating calculation 5 times using 10 to 10,000 random draws. To this end, we derived the standard deviation associated with the 5 repeated calculation of *K*_i,max_.

From the optimal number of random draws, *K*_i,max_ value measured by our method was compared to the reference *K*_i_ value derived from Patlak analysis considering the voxel with the maximal intensity. The same comparison was done with Hunter and Barbolosi methods (Barbolosi-Bl or Barbolosi-Im).

To compute reference *K*_i_, using the Patlak approach, the early 30 min PET acquisition centered over the heart is required, along with the late 15 min PET acquisition centered over the lesion acquired 80–90 min post-injection and 5 venous blood samples (each 3 min).

Hunter and Barbolosi original methods (Barbolosi-Bl) required the late 15 min acquisition reconstructed as 5 × 3 min acquisitions associated with venous blood samples. Whole blood activity was measured using a high energy dedicated Gamma Counter.

Barbolosi-Im and ParaPET approaches only require a late dynamic PET acquisition centered over the lesion without any blood sampling.

To assess the agreement between the Patlak *K*_i_ and *K*_i,max_ measured by all methods, Bland-Altman ratios (*K*_i,max_/Patlak *K*_i_ as a function of averaged value of *K*_i,max_ and Patlak *K*_i_) and intraclass correlation coefficients (ICC) were calculated. The statistical comparison of the methods was assessed by a Wilcoxon paired test. The 95% limits of agreement on the Bland-Altman plots were expressed as a percentage of the mean *K*_i_ ratios.

Considering the whole set of lesions, *K*_i,max_ obtained with ParaPET was compared to the lesion SUV_max_ index measured on a 3 min acquisition at time *t*_k = 1_. The agreement was evaluated using Pearson correlation coefficient and its statistical significance by the *p* value.

We also investigated the influence of *K*_i,max_ and SUV_max_ in the tumour response evaluation, considering the Δ*K*_i,max_ and ΔSUV_max_ (unitless) between the baseline and per-therapeutic exams:

$$ \Delta {K}_{\mathrm{i},\max }=\left({K}_{\mathrm{i},42\mathrm{Gy}}-{K}_{\mathrm{i},\mathrm{baseline}}\right)/ Ki,\mathrm{baseline} $$

$$ {\Delta \mathrm{SUV}}_{\mathrm{max}}=\left({\mathrm{SUV}}_{\max, 42\mathrm{Gy}}\hbox{-} {\mathrm{SUV}}_{\max, \mathrm{baseline}}\right)/{\mathrm{SUV}}_{\max, \mathrm{baseline}} $$

Bland-Altman plots and ICC were calculated. The statistical comparison was assessed by a Wilcoxon signed-rank test.

## Results

Table 1 gives the patient demographics. To date, 31 patients were included in this on-going clinical trial, yielding a total of 52 parametric PET sequences (31 baseline and 18 per-therapeutic). The total number of lesions studied is 82 (51 in baseline, 31 in per-therapeutic exams).

**Table 1:** Patient demographics and characteristics

Number of patients		31
Number of exams		52
Male/female (%)		72 /28
Age (years)		63.1 ± 8.3
Histology	ADK	42%
	SCC	48%
	LCLC	10%
Location	Right	
	Upper	39%
	Middle	13%
	Lower	10%
	Left	
	Upper	29%
	Lower	10%
Average failure rate of blood sampling	No samples or unusable	35%
	1 to 3 samples	13%
	≥ 4 samples	52%

Among the 52 exams realised, we were able to obtain at least four blood samples for only 52% of our exams. This result clearly shows how difficult it is to implement methods requiring blood samples. Therefore, we were finally only able to compare the ParaPET methodology with Patlak, Hunter and Barbolosi for 41/82 lesions.

Regarding the comparison between ParaPET images and standard PET images, 82 lesions were included and 25 lesions with a complete baseline and per-therapeutic 42 Gy sequences.

**Fig. 4:**
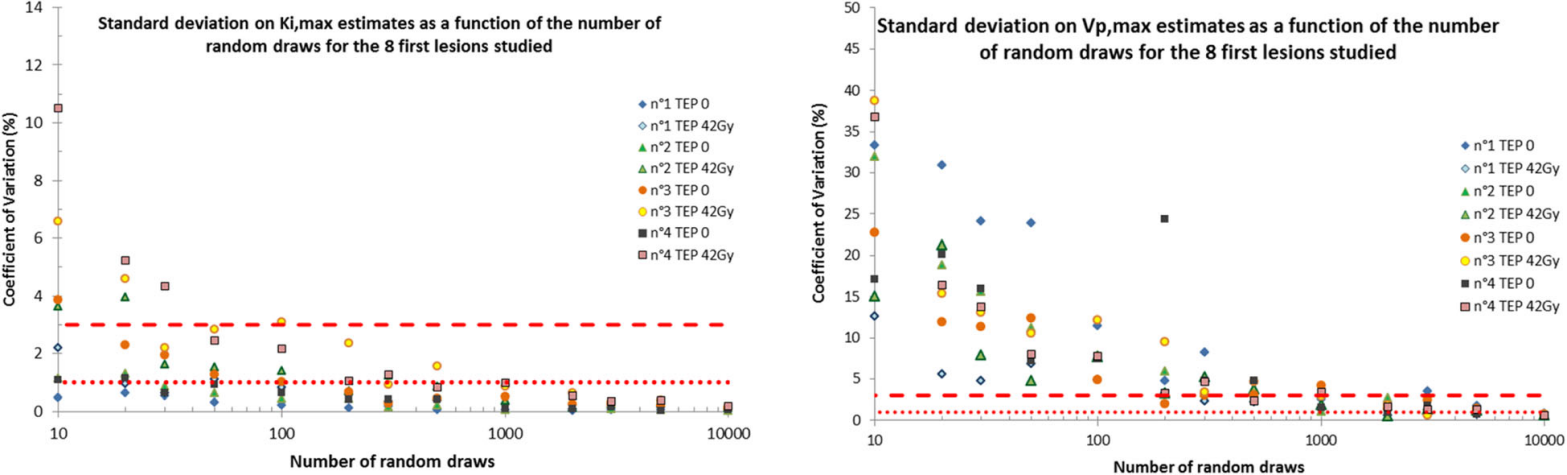
Evaluation of the robustness of our proposed method; *K*_i,max_ (left fig.) and *V*_p,max_ (right fig.) estimates using 10 to 10,000 random draws for 8 lesions. Each calculation was repeated five times to derive the standard deviation on *K*_i,max_ estimates. The coefficients of variation of 1% and 3% are represented by the dotted and the dashed lines respectively

Fig. 4a, b shows the results of the influence of the number of random draws on the robustness of the estimation of *K*_i,max_ and *V*_p,max_ values for the eight first lesions studied. The same behaviour was found with the other lesions. The method clearly presents a fast convergence to a stable solution for *K*_i_ and *V*_p_. Considering a maximum coefficient of variation (CV) of 3%, our results show that 1000 random draws are required to correctly estimate *K*_i,max_ and *V*_p_.

**Fig. 5:**
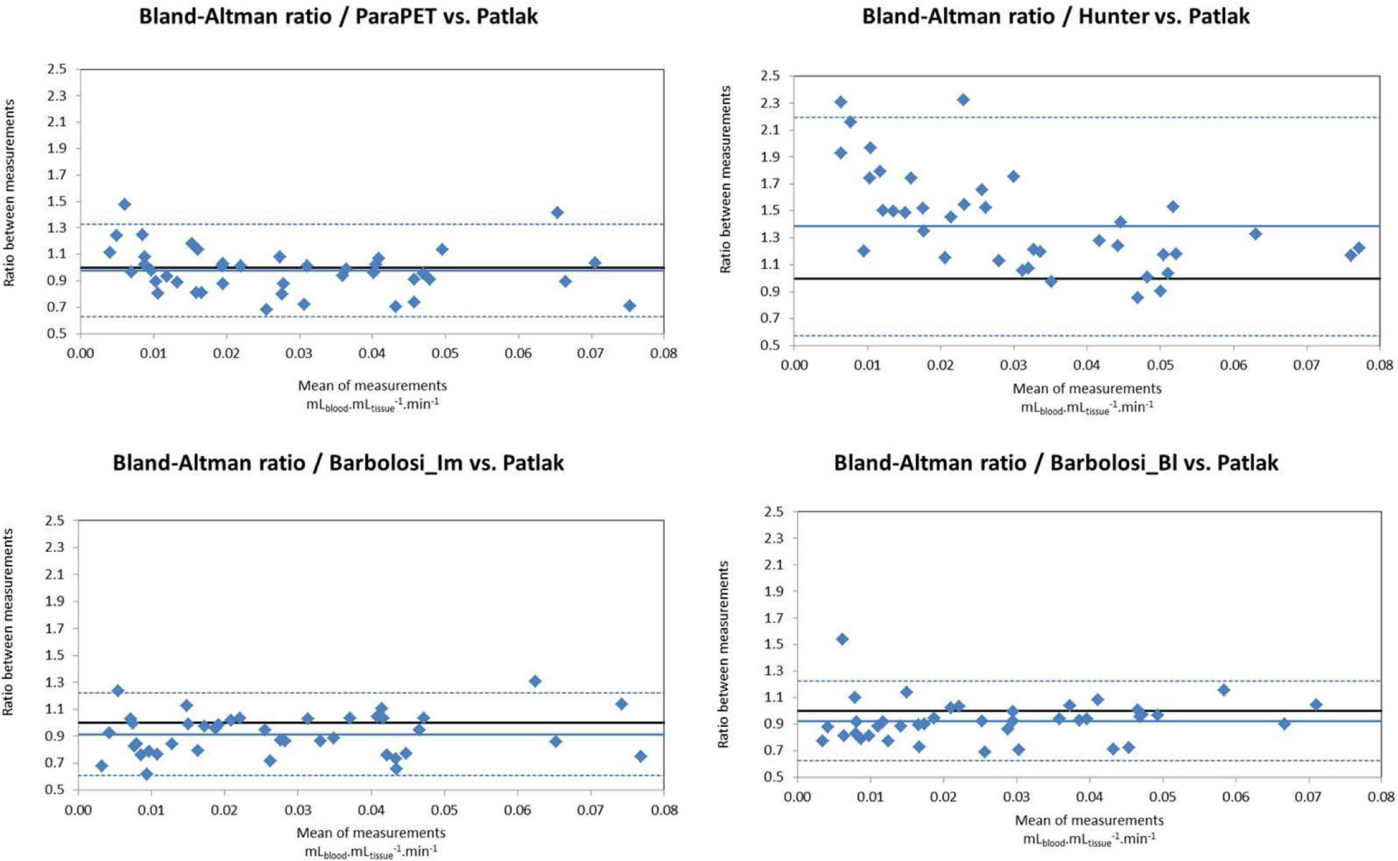
Bland-Altman ratios between the Patlak *K*_i,max_ value and *K*_i,max_ values derived from other approaches. Results for ParaPET (upper left), Hunter’s method (upper right), Barbolosi-Im method (bottom left) and Barbolosi-Bl method (bottom right). All these values correspond to the same voxel position. The mean ratio and the 1.96 SD limits are represented by the solid and the dashed lines respectively

Regarding the estimation of the *K*_i,max_ index, all methods show a good correlation with Patlak (ICC of 0.905 for Hunter and > 0.935 for the other approaches). Compare to Patlak, the median relative difference and associated range (median; [min;max]) in *K*_i,max_ estimates were not statistically significant (Wilcoxon test) for ParaPET (− 3.0%; [− 31.9%;47,3%]; *p* = 0.08) but statistically significant for Barbolosi-Bl (− 8.0%; [− 30.8%;53.7%]; *p* = 0.001), Barbolosi-Im (− 7.9%; [− 38.4%;30.6%]; *p* = 0.007) or Hunter (32.8%; [− 14.6%;132.2%]; *p* < 10–5). Figure 5 displays the Bland-Altman ratios between the Patlak value of *K*_i,max_ and the values found with ParaPET, Barbolosi-Bl and Barbolosi-Im. This figure shows more clearly the large bias observed with the Hunter’s methodology, and its dependence with the *K*_i_ amplitude that was not observed for Barbolosi or ParaPET methods. The 95% limits of agreement are comparable for ParaPET (34.7%), Barbolosi-Bl (30.1%) and Barbolosi-Im (30.8%), but much lower than Hunter’s (81.1%) and do not highlight the superiority of ParaPET nor Barbolosi’s approaches.

**Fig. 6:**
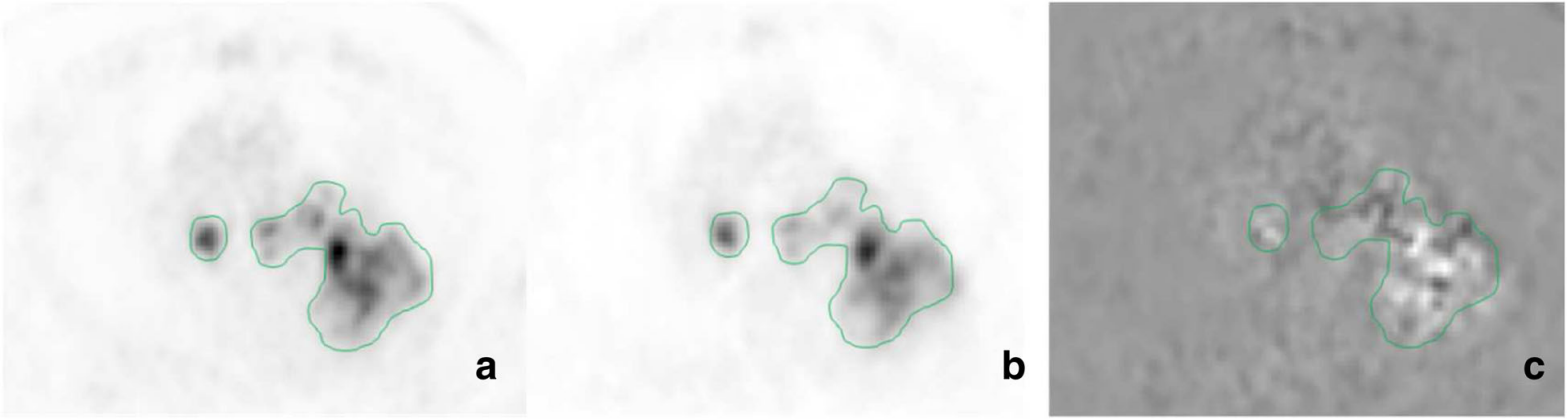
Example of SUV (**a**), *K*_i_ (**b**), and subtraction (**c** = **a**–**b**) PET images generated by our approach for a patient with multiple lesions. The subtraction image (**a**–**b**) is obtained after the normalisation of the images considering the total number of counts measured in the slice. The external contours of the lesions are represented by the solid green line

**Fig. 7:**
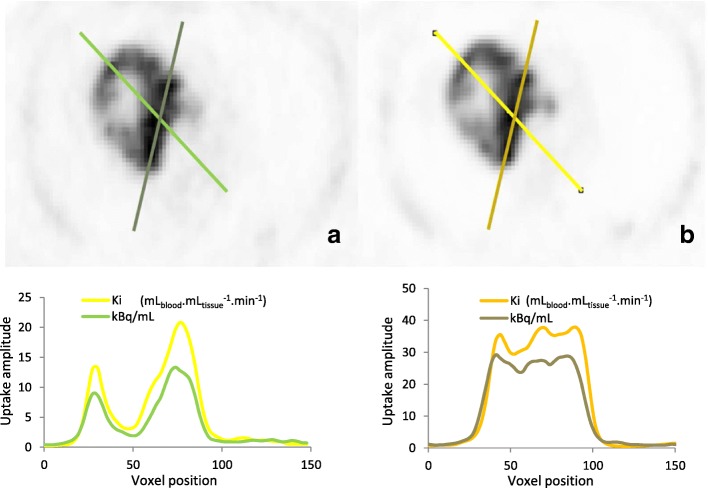
Example of profiles drawn through the lesion of the SUV (**a**) and *K*_i_ (**b**) PET images; *K*_i_ and SUV images were normalised to the background activity of the SUV image

Figs. 6 and 7 show examples of SUV and *K*_i_ PET images generated by our approach. For comparison purpose, *K*_i_ and SUV images were normalised to the total number of counts measured in the slice for Fig. 6, or in the background activity of the SUV image for Fig. 7. These images are highly correlated as it was previously reported [17], even if small differences can be observed at the voxel level as it can be seen in the subtraction image (see Fig. 6).

As suggested by other groups, the *K*_i_ images present a higher image contrast, as it can be seen on the profiles drawn through the lesion on Fig. 7, especially within the necrotic region of the lesion. In this example, the contrast between lesion maximum uptake and the necrotic region was improved from 5.1 in the SUV image to 5.7 in the *K*_i_ image.

**Fig. 8:**
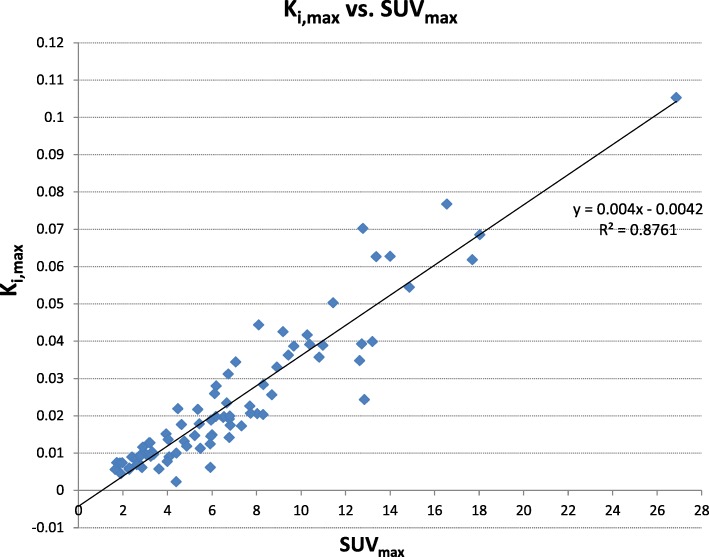
Regression line between the ParaPET *K*_i,max_ value and SUV_max_ values

The correlation plot between the SUV_max_ and *K*_i,max_ index for the 52 examinations is displayed on Fig. 8. This figure clearly highlights the strong correlation between the 2 indexes.

**Fig. 9:**
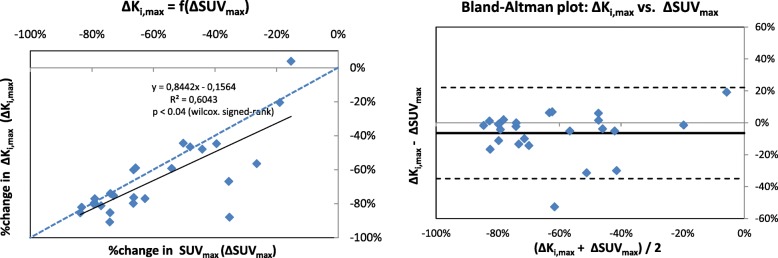
Regression line and Bland-Altman plots between the ParaPET Δ*K*_i,max_ value and ΔSUV_max_ values. For each lesion, *K*_i,max_ and SUV_max_ were measured at the same voxel position. On the regression graph, the dashed line is the line of equality. On the Bland-Altman plots, the mean difference and the 1.96 SD limits are represented by the solid and the dashed lines respectively

In complement, Fig. 9 shows the difference between Δ*K*_i,max_ and ΔSUV_max_ index which represent the variation of FDG uptake before (J0) and during radiochemotherapy (J42 Gy). In the 25 lesions imaged before and during the radio-chemotherapy, the Δ*K*_i,max_ is statistically lower than the ΔSUV_max_ (*p* < 0.02 Wilcoxon one-tailed test). The linear relation between those indexes is given by Δ*K*_i,max_ = 0.84 ΔSUVmax–0.15 with a median additional variation of − 2.3% [− 52.6%; + 19.1%].

## Discussion

This paper proposes a new non-invasive methodology, called ParaPET, allowing the determination of 3D map of FDG kinetic parameters. Our approach is adapted to clinical routine and requires only a late dynamic PET acquisition of a limited duration corresponding to 15 min, performed after the whole-body PET/CT acquisition, 80 min after FDG injection in our evaluation. Furthermore, blood samples are not necessary to perform our method.

In FDG PET, kinetic parameters are estimated using the Patlak graphical analysis as a reference, according to the European Organization for Research and Treatment of Cancer (EORTC) [18]. Although Patlak analysis is robust, the original method has three major practical limitations: the need for continuous blood sampling, a 1-h dynamic acquisition and the assumption of irreversible trapping. Several alternatives have been proposed, but they still require a long dynamic PET acquisition and the inclusion of blood compartments in the field of view [19, 20]. In this study, the implementation of Patlak analysis is performed using a shortened dynamic FDG PET protocol based on an early 30 min dynamic PET acquisition starting from the injection of FDG and a late 15 min PET sequence acquired 80 min post injection. The shape of the FDG blood activity concentration function *C*_*p*_(*t*) is derived from the early dynamic PET acquisition and five late blood measurements.

In the literature, refined quantitative methods based on the Markov chain model [21] or direct 4D parametric PET images using expectation-maximisation [15] or penalised maximum-likelihood [14] reconstruction algorithms have been proposed allowing accurate calculation of kinetic parameters. Although these kinetic modelling methods are powerful, their complexity, the slow convergence rate of reconstruction algorithms and the need of long PET acquisition have prevented their clinical adoption.

Recently, as an alternative to Patlak approach, Barbolosi et al. [13] proposed a methodology for the determination of kinetic parameters such as the metabolised and unmetabolised FDG fractions. This method, evaluated on paragangliomas [13] and on NSCLC [22], takes into account the variability of activity concentration measurements. However, three major limitations have to be highlighted. First, the method was designed to compute global kinetic parameter values for the whole lesion, whereas parametric images at a voxel level might provide additional quantitative information. Second, the determination of $\overline{C_{\mathrm{FDG}}\left({t}_k\right)}$ and its variance ${\sigma}_{\mathrm{FDG},{t}_k}^2$ is questionable, since values are derived from the maximum FDG activity concentration within three consecutive 2D slices surrounding the maximum activity concentration of the whole lesion (see Fig. 1). Third, this approach is invasive requiring several blood samples. We have faced these drawbacks by proposing a non-invasive methodology allowing the determination of FDG kinetic parameters at the voxel level, based on a new error model of measurement and a late IDIF.

As an alternative to blood samples, FDG activity concentration in blood can be extracted from dynamic PET images [15]. Regarding the determination of $\overline{C_{\mathrm{FDG}}\left({t}_k\right)}$ and its variance ${\sigma}_{\mathrm{FDG},{t}_k}^2$, our approach assumes that the FDG burden remain stable during each 3 min listmode datasets used to derived the mean FDG uptake and its associated variance. Our approach is naturally limited to lesions presenting an irreversible trapping since it is based on the Patlak kinetic model.

The validation of our methodology was carried out by taking the Patlak method as a reference rather than the full compartmental analysis. Unfortunately, the health status of the patients included in this protocol was not compatible with the realisation of 60 min dynamic acquisition required for the full compartmental approach. This also explains the low blood collection rate that we have been able to obtain in patients with severe reduction of their venous capital, especially after few weeks of radio-chemotherapy.

In the implementation of Barbolosi’s, Hunter’s and Patlak approaches, whole blood was used instead of plasma activity concentrations. This simplification was evaluated on the first four patients (four baseline and three per-therapeutic exams), comparing the measurements of the FDG concentration in the total blood or in the plasma (extracted by a 3000 rotation/min during 10 min). Seventy samples were compared (7 exams × 5 samples divided into 2 sub-samples). The relative difference was considered negligible (mean relative difference and associated standard deviation of 0.60 ± 1.98%) and whole blood was finally used in the rest of the study.

The evaluation of the number of random draws showed that 1000 random draws are necessary to compute *K*_i,max_ with a CV below 1% and *V*_p,max_ with a CV below 3%.

In comparison to Hunter and Barbolosi methods, we showed that our approach presents the lowest relative bias in the estimation of the reference *K*_i,max_. However, in the Bland-Altman ratio, the 95% limits of agreement remain too elevated (LoAs > 30%) to conclude in the replacement of the Patlak approach by any of the four methods. Nevertheless, even if our method does not seem to differ from Barbolosi’s methods in terms of median error and range, it allows avoiding blood samples and requires only a late acquisition, making its clinical use possible. It also provides a map of *K*_i_ values, which is not possible with the other methods evaluated in this work.

The Bland-Altman is a graphical representation of the agreement between two methods or evaluators. It does not give any *p* value to assess the statistical significance of this agreement. We then decided to add the evaluation of the statistical difference between Patlak *K*_i_ values and the *K*_i_ estimates from the other approaches using a Wilcoxon paired test.

We assumed that the noise characteristic of our reconstructed image is correctly estimated using a simple resampling of our listmode data. The best approach would have been to derive the standard deviation of tumour absorption using a conventional bootstrap approach, generated by randomly drawing, with replacement, prompt and random events [23]. We then consider our solution as a particular case of a bootstrap approach. Implementing a real bootstrap approach was not possible for this study but will be integrated as an evolution of our methodology.

The large variability of the errors in the estimation of *K*_i_ with ParaPET or Barbolosi’s methods might be due to the relatively short time used to derive the *K*_i_ values (only 15 min). The inclusion of the whole-body acquisition could help to reduce the error and probably increase the accuracy of our methodology in comparison with Patlak, computed with the 0–30 min and the 80–95 min post-injection datasets.

Hunter’s method presents the highest mean error in the estimation of *K*_i_. This method was developed to be applied at 55 min post-injection. Since the blood activity is reduced at later time (80 min PI) and given that Hunter’s method neglects the unmetabolized part of the FDG (i.e. *V*_p_ = 0), applying Hunter’s method at later time may have a negative impact on its *K*_i_ estimates. Whatever the method, using late acquisitions with very low blood concentrations is very challenging since we derive the actual blood activity concentration, and the measure variability from those late series. However, due to practical consideration, using sooner series was not possible for this study. Nevertheless, the results even not perfect are encouraging and further improvements are still required. Here again, the inclusion of the whole-body acquisition would help to improve our approach.

As it was underlined in other papers [7, 24, 25], the *K*_i_ index, even highly correlated with the SUV values, gives additional information. When looking at the tumour response with therapy, tumour response based on changes in *K*_i,max_ may thus be different than those measured with SUV_max_ values. The variation in *K*_i,max_ is statistically higher than the variation in SUV_max_ values. Considering the EORTC or PERCIST criteria, the tumour response could be modified considering the Δ *K*_i,max_ instead of the ΔSUV_max_ value. For clinical trials, this could imply that use of SUV for assessment of radio-chemotherapy may under- or overestimate treatment efficiency compared with parametric analysis.

In addition, as shown in Fig. 6, the *K*_i_ and SUV images are not completely identical. The use of parametric images (*K*_i_, *V*_p_) for the determination of predictive and/or prognostic values based on radiomic features will remain to be investigated.

The main limitations of this study is the restriction of the validation of our 3D approach to *K*_i,max_ corresponding to the maximum value of the net influx rate within the entire lesion instead of a full 3D evaluation.

Our approach is adapted to clinical routine since it only requires a dynamic PET acquisition of a limited duration corresponding to 15 min, performed 80 min after FDG injection in our evaluation. To date, our approach is limited to one bed position. In the next few years, the new cristal-SiPM PET system, with larger field of view and increased sensitivity, will probably allow to implement the methodology for larger field of view.

Another limitation of our approach, to be applied in clinical routine, is the time needed to obtain parametric 3D maps. Efforts could be made to optimise the calculation time by using a parallelized process.

A PET system manufacturer recently proposes an automatic non-invasive Patlak whole-body acquisition in their scanner. Their approach even if it requires a 60-min acquisition, brings Patlak analysis back to the clinical applications. In that context, our approach could be a practical alternative to derive Patlak images only requiring a 15-min PET acquisition.

In a further step, the clinical relevance of our approach, already suggested in the paper from Padovani et al. [22] will be evaluated with the NSCLC patients included into the on-going ParaPET clinical trial (NCT 02821936). The aim of this trial is the assessment of the predictive and prognostic value of biomarkers extracted from the 3D map of kinetic parameters in the progression-free and overall survival. The results presented here have allowed us to validate our approach for the evaluation of prognostic and predictive values of parametric images.

Another major perspective of our approach is the evaluation of response to immunotherapy treatment. Indeed, so far, conventional PET imaging has shown its limits in the detection and interpretation of the tumour pseudo-progression due to an inflammatory process. Parametric imaging offers new insights into the use of FDG-PET for this purpose.

## Conclusion

The main contribution of this study is the development of a non-invasive voxel-based approach for the determination of 3D parametric images of kinetic parameters of FDG uptake, integrating a new error model of PET measurement and a late IDIF. Our approach is not yet ready to replace the Patlak approach, and further improvements are still required. Nevertheless, ParaPET remains a promising approach, offering a non-invasive alternative to methods based on multiple blood samples and only requiring a simple 15 min listmode acquisition. It provides a viable alternative for the voxel-based estimation of the net influx rate *K*_i_, giving access to additional heterogeneity information which could be used for radiomic purpose.

We confirmed in a population of patient treated by radio-chemotherapy that our ParaPET approach yields *K*_i_ indexes non-statistically different than Patlak *K*_i_. As previously described, changes in SUV_max_ during the treatment is not correlated with the changes measured from *K*_i_ indexes.
